# A retrospective cohort study on the clinical outcomes of patients admitted to intensive care units with dysnatremia

**DOI:** 10.1038/s41598-023-48399-5

**Published:** 2023-12-01

**Authors:** Pauline Yeung Ng, Regina Yui Ting Cheung, April Ip, Wai Ming Chan, Wai Ching Sin, Desmond Yat-Hin Yap

**Affiliations:** 1 Critical Care Medicine Unit, School of Clinical Medicine, The University of Hong Kong, Hong Kong SAR, China; 2 Department of Adult Intensive Care, Queen Mary Hospital, Hong Kong SAR, China; 3 Department of Medicine, The University of Hong Kong, Hong Kong SAR, China; 4 Department of Medicine, Queen Mary Hospital, The University of Hong Kong, Room 301, 3/F New Clinical Building, 102 Pokfulam Road, Hong Kong SAR, China

**Keywords:** Acid, base, fluid, electrolyte disorders, Health care economics, Outcomes research

## Abstract

With evolving patient characteristics and patterns of ICU utilization, the impact of dysnatremias on patient outcomes and healthcare costs in the present era have not been well studied. Patients ≥ 18 years admitted to the ICUs in public hospitals in Hong Kong between January 2010 and June 2022 and had at least one serum sodium measurement obtained within 24 h prior to or following ICU admission were stratified into normonatremic (135-145 mmol/L), hyponatremic (< 135 mmol/L) and hypernatremic (> 145 mmol/L) groups. A total of 162,026 patients were included—9098 (5.6%), 40,533 (25.0%) and 112,395 (69.4%) patients were hypernatremic, hyponatremic and normonatremic at the time of ICU admission, respectively. The odds of patients with hypernatremia and hyponatremia dying in the ICU were 27% and 14% higher (aOR 1.27, 95% CI 1.19–1.36 and aOR 1.14, 95% CI 1.08–1.19, respectively; P < 0.001 for both), and 52% and 21% higher for dying in the hospital (aOR 1.52, 95% CI 1.43–1.62 and aOR 1.21, 95% CI 1.17–1.26, respectively; P < 0.001 for both] compared with those with normonatremia. Patients with dysnatremia also had longer ICU length of stay (LOS), hospital LOS, and higher healthcare costs than the normonatremic group. Dysnatremias at ICU admission were associated with increased ICU and in-hospital mortality and overall healthcare burden.

## Introduction

Dysnatremias are common electrolyte disorders in the Intensive Care Unit (ICU) setting. The prevalence of dysnatremias has been reported to be up to 24.6% upon ICU admission, depending on the type of dysnatremias, severity of dysnatremias, and patient population^1^. The presence of serum sodium (Na) abnormalities prior to or following ICU admission is clinically important, as both hypernatremia and hyponatremia have been denoted as independent predictors for increased ICU mortality^2,3^. The management of dysnatremias, especially severe ones, are challenging as overzealous correction may lead to serious neurological sequelae such as osmotic demyelination or cerebral edema^4^.

Although the association between dysnatremias and clinical outcomes have been studied in different clinical scenarios, the impact of dysnatremias on patient outlook after ICU admission in the recent era have not been well characterized. Over the past two decades, there have been temporal changes in the pattern of ICU utilization, owing to the ageing population and evolving patient characteristics, advances in the treatment for medical and surgical conditions, e.g. novel anti-cancer therapies, advanced life support systems and surgical techniques, as well as the emergence of new disease entities, e.g. COVID-19 disease^5^. The overall number of ICU admissions have also increased by 25% in the last 10 years^6^. Based on these observations, we postulate that the clinical epidemiology and impact of dysnatremias have changed considerably in critically ill patients admitted to the ICU. Indeed, a shift in the incidence from hyponatremia to increasing hypernatremia in the ICU has been reported in one study^7^. Furthermore, existing literature often focuses on ICU-acquired dysnatremias or certain ICU sub-cohorts with limited patients numbers, rendering the results less generalizable^2,8^. For example, important consequences of dysnatremias such as adverse neurological sequelae are largely studied in neurosurgical patients but not in other ICU patients with non-neurological conditions^4^.

In view of these knowledge gaps, we used data from a territory-wide electronic health system over a 12-year period to evaluate the impact of dysnatremias on mortality, neurological complications, and healthcare costs in adult patients admitted to the ICU. The hypothesis was that dysnatremias at ICU admission are associated with increased ICU mortality, worse neurological outcomes, and increased healthcare costs in mixed surgical and medical ICU patients.

## Methods

### Study design and data source

This retrospective cohort study used data from a territory-wide electronic health record system in Hong Kong from 1st January 2010 to 30th June 2022. The study conforms with the principles outlined in the Declaration of Helsinki and was approved by the Institutional Review Board of the University of Hong Kong/Hospital Authority Hong Kong West Cluster (HKU/HA HKW IRB) (IRB Reference Number: UW 22-650) with a waiver of signed informed consent.

### Study population

Patients who were ≥ 18 years at the time of ICU admission and had at least one serum Na measurement obtained within 24 h prior to or following ICU admission were included. Patients with missing Acute Physiology and Chronic Health Evaluation IV (APACHE IV) predicted risk of death or missing creatinine measurement within 24 h prior to or following ICU admission were excluded from this study.

### Study exposure/definitions

Patients were stratified according to their closest serum Na measurement obtained within 24 h prior to or following ICU admission into the normonatremic (serum Na 135–145 mmol/L), hyponatremic (serum Na < 135 mmol/L), and hypernatremic (serum Na > 145 mmol/L) groups, respectively.

### Study outcomes

The co-primary outcomes were ICU and in-hospital mortality. Secondary outcomes included adverse neurological outcomes, hospital discharge destination, ICU length of stay (LOS), hospital LOS, and healthcare costs. An adverse neurological outcome was defined by a clinical surrogate of requiring one or more computed tomography or magnetic resonance imaging scans of the brain performed between 24 h after ICU admission and hospital discharge. Hospital discharge destination was categorized into “death”, “home”, “transfers” which included patients who were transferred to convalescent beds, or “others” which included patients who did not have documented hospital discharge destination information. Healthcare costs were estimated using the bed costs with respective to the number of bed days from ICU admission to hospital discharge. Bed costs were based on 4 types of bed status provided in public hospitals in Hong Kong—intensive care bed (HK$ 24,400/day, i.e. US$ 3128/day), acute general or convalescent bed (HK$ 5100/day, i.e. US$ 654/day), and psychiatric bed (HK$2340/day, i.e. US$ 300/day)^9^.

### Data collection

Patients’ baseline characteristics and clinical variables were collected using the clinical data analysis and reporting system (CDARS), a central de-identified data repository comprising electronic health records from all public hospitals in Hong Kong. Previous data validation for use in cohort studies has shown high coding accuracy^10^. Patients’ demographics, diagnoses, hospitalization, prescriptions, laboratory results and outcomes were retrieved. All data were de-identified to ensure patients’ privacy and confidentiality. Comorbidities were identified on the basis of routinely recorded codes from the International Classification of Diseases, 9th Revision, Clinical Modification (ICD-9-CM) (see Supplementary Sect. 1.1 online).

### Statistical analysis

Categorical variables were described as frequencies with percentages (%), and continuous variables as median with interquartile range (IQR). Categorical variables were compared with Fisher-exact test or Pearson’s chi-squared test, as appropriate. ANOVA test was used to compare the differences among the exposure groups. Demographics and clinical variables that were significantly different between the exposure groups with a P-value < 0.05 and other confounders selected a priori were included in multivariable logistic regression. The variables including APACHE-IV predicted risk of death, age, sex, creatinine level obtained within 24 h prior to or following ICU admission, cardiovascular diseases, respiratory diseases, kidney diseases, liver diseases, cerebrovascular diseases, diabetes mellitus (DM), dementia, immunosuppressive therapy, presence of COVID-19 infection, emergent ICU admission, and surgical ICU admission were included in the final multivariable logistic regression model. All variables included in the regression model were tested for collinearity.

The association between exposure groups and the outcomes of length of stay and medical costs were examined using multivariable adjusted negative binomial regression and generalized linear model, respectively. A local polynomial smooth plot was used to model the changes in plasma Na level over the first 7 days of ICU admission by fitting the moving weighted average of serum Na levels for all 3 groups (details are included in Supplementary Method). All analyses were performed with two-tailed tests and a P-value < 0.05 was considered statistically significant. All analyses were done using STATA MP, version 16.1.

### Subgroup analysis

Subgroup analyses were performed to assess the effect of serum Na on ICU mortality in the surgical and medical cohorts and patients admitted to ICU for neurological and non-neurological conditions. Detailed definitions of subgroups are included in Supplementary Sect. 1.2).

### Sensitivity analysis

Sensitivity analyses included, first, redefining the cut-off value for dysnatremias – normonatremia (serum Na level 130–150 mmol/L), hyponatremia (serum Na level < 130 mmol/L), and hypernatremia (Na level > 150 mmol/L)^8^. Second, the effect of hyperglycaemia on serum Na was accounted for by calculating corrected serum Na levels using the following formula: Corrected [Na] = Serum [Na] + 0.4 x (Paired blood glucose – 5.5 mmol/L)^11^. Paired blood glucose level was defined as the blood glucose measurements taken within 3 h prior to or following the corresponding serum Na measurement. Patients were reclassified into three groups according to the corrected Na, and ICU mortality was compared between groups.

### Exploratory analysis

Subjecting to observed heterogeneity between subgroups of patients with and without DM, interaction analysis was performed by including the interaction term “dysnatremia x diabetes” to the main regression model and re-examining the association with ICU mortality.

### Ethics approval and consent to participate

This study was approved by the Institutional Review Board of the University of Hong Kong/Hospital Authority Hong Kong West Cluster (HKU/HA HKW IRB) (IRB Reference Number: UW 22-650) with a waiver of signed informed consent. This study complies with the Declaration of Helsinki 1975 and its later amendments.

## Results

### Study population and patient characteristics

**Figure 1:**
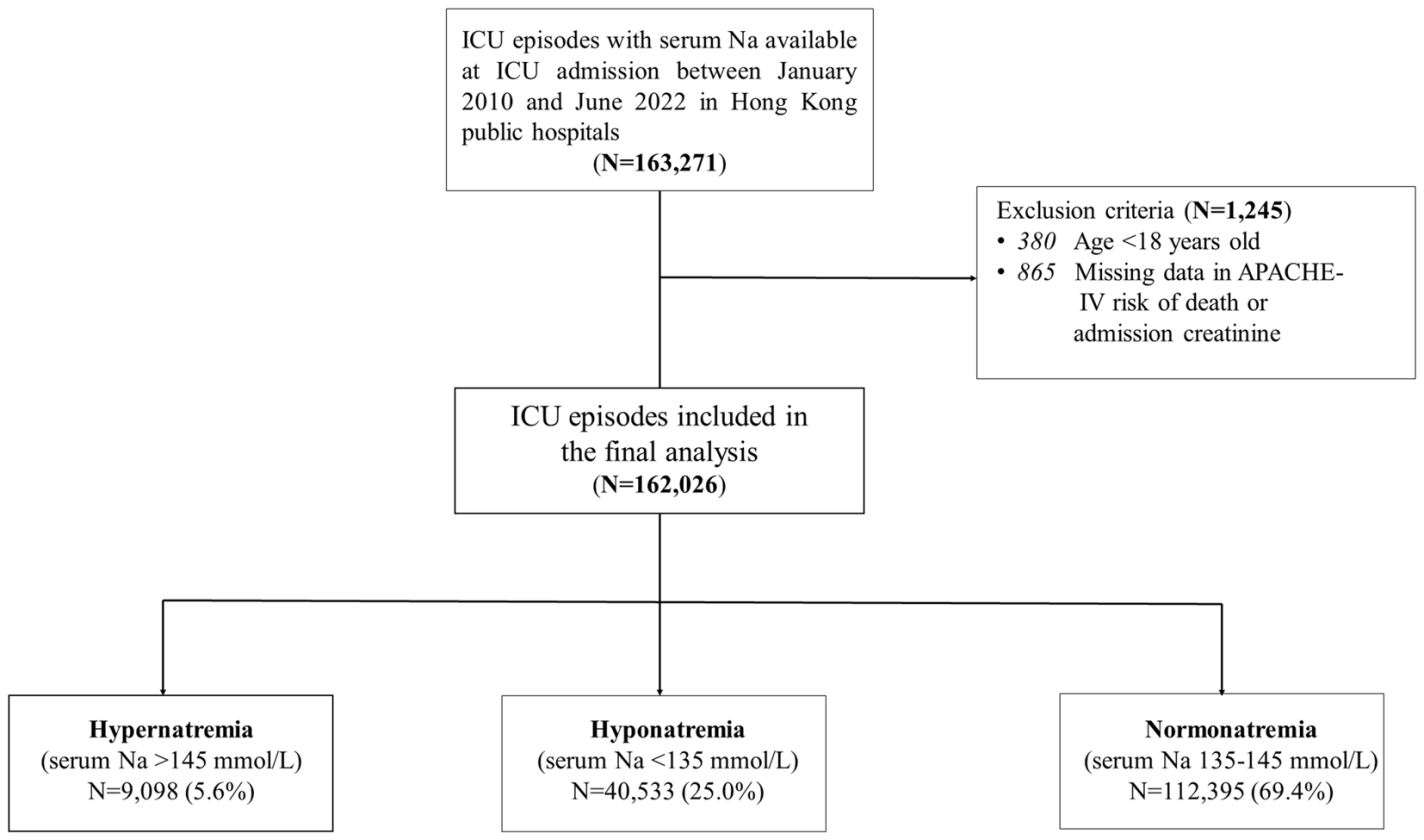
Study flow. A total of 163,271 ICU episodes with serum Na results available were identified between January 2010 and June 2022. After excluding 1245 (0.8%) episodes which had met exclusion criteria, a total of 162,026 ICU episodes were included in the final analysis. *APACHE IV* acute physiology and chronic health evaluation IV, *ICU* intensive care unit, *Na* sodium.

A total of 163,493 patients were admitted to the ICUs in public hospitals in Hong Kong between 1st January 2010 and 30th June 2022. After excluding patients with missing data and those younger than 18 years, a total of 162,026 patients were included in the final analysis. The median age was 64 (52–74) years, and 99,804 (61.6%) patients were male. The median APACHE IV predicted risk of death was 0.12 (0.04–0.33). There were 70,080 (43.3%) and 91,946 (56.8%) surgical and medical ICU patients, respectively. A total of 78,453 (48.4%) patients had cardiovascular diseases, 18,534 (11.4%) had kidney diseases, 21,329 (13.2%) had cerebrovascular diseases, and 34,278 (21.2%) had DM. The study flow and baseline characteristics are shown in Fig. 1 and Table 1, respectively.

**Table 1:** Baseline characteristics of patients with hypernatremia, hyponatremia and normonatremia at the time of ICU admission.

	Hypernatremia	Hyponatremia	Normonatremia	P-value
	N = 9098	N = 40,533	N = 112,395	
Gender, male	5609 (61.7%)	24,787 (61.2%)	69,408 (61.8%)	0.10
Age, years	64.8 (54.0–75.8)	65.3 (54.5–75.7)	63.2 (51.5–73.9)	< *0.001*
APACHE IV risk of death	0.3 (0.1–0.7)	0.2 (0.1–0.4)	0.1 (0–0.3)	< *0.001*
Reasons for ICU admission				< *0.001*
Sepsis	1891 (20.8%)	9239 (22.8%)	13,739 (12.2%)	
Gastrointestinal	1881 (20.7%)	5886 (14.5%)	22,786 (20.3%)	
Cardiovascular	1245 (13.7%)	5895 (14.5%)	18,974 (16.9%)	
Respiratory	1036 (11.4%)	4821 (11.9%)	15,419 (13.7%)	
Metabolic	991 (10.9%)	5810 (14.3%)	6523 (5.8%)	
Neurological	911 (10.0%)	2524 (6.2%)	16,801 (14.9%)	
Renal/genitourinary	441 (4.8%)	3401 (8.4%)	4973 (4.4%)	
Trauma	347 (3.8%)	1067 (2.6%)	7078 (6.3%)	
Musculoskeletal/skin	219 (2.4%)	1462 (3.6%)	3440 (3.1%)	
Gynecological	101 (1.1%)	296 (0.7%)	2350 (2.1%)	
Hematological	35 (0.4%)	131 (0.3%)	311 (0.3%)	
Types of ICU admission				
Emergent	7935 (87.2%)	36,534 (90.1%)	80,990 (72.1%)	< *0.001*
Surgical	3195 (35.1%)	11,321 (27.9%)	55,564 (49.4%)	< *0.001*
Comorbidities^1^				
Cardiovascular diseases	4034 (44.3%)	21,258 (52.4%)	53,161 (47.3%)	< *0.001*
Malignancies	2133 (23.4%)	9348 (23.1%)	29,332 (26.1%)	< *0.001*
Diabetes mellitus	1733 (19.0%)	12,053 (29.7%)	20,492 (18.2%)	< *0.001*
Cerebrovascular diseases	1185 (13.0%)	4731 (11.7%)	15,413 (13.7%)	< *0.001*
Kidney diseases	1028 (11.3%)	9115 (22.5%)	8391 (7.5%)	< *0.001*
Respiratory diseases	754 (8.3%)	3370 (8.3%)	10,385 (9.2%)	< *0.001*
Immunosuppressed	517 (5.7%)	2703 (6.7%)	5507 (4.9%)	< *0.001*
Liver diseases	389 (4.3%)	1112 (2.7%)	2409 (2.1%)	< *0.001*
Dementia	103 (1.1%)	250 (0.6%)	677 (0.6%)	< *0.001*
COVID-19	85 (0.9%)	725 (1.8%)	750 (0.7%)	< *0.001*
Laboratory data at ICU admission				
Serum sodium, mmol/L	148 (146.1–151.1)	131.3 (128.0–133.2)	139 (137.0–141.0)	< *0.001*
Serum creatinine, mg/dL	1.4 (0.9–2.3)	1.3 (0.8–3.2)	0.9 (0.7–1.4)	< *0.001*
Serum albumin, g/L^2^	26 (20.0–32.4)	28.6 (23.0–34.3)	31 (25.0–36.9)	< *0.001*
Premorbid medications				
Diuretics^3^	3303 (36.3%)	15,468 (38.2%)	30,961 (27.5%)	< *0.001*
Antidiuretics^4^	645 (7.1%)	2083 (5.1%)	2529 (2.3%)	< *0.001*
Corticosteroids^5^	3624 (39.8%)	14,017 (34.6%)	32,640 (29.0%)	< *0.001*

All data were presented as frequency (percentage) or median (interquartile range (IQR)) unless specified.

*APACHE IV* acute physiology and chronic health evaluation IV, *ICU* intensive care unit.

^1^Defined according to acute physiology and chronic health evaluation IV or international classification of disease clinical modification.

^2^44,312 (27.3%) patients did not have serum albumin measurements at ICU admission.

^3^Diuretics included hydrochlorothiazide, indapamide, dyazide, moduretics, metolazone, frusemide, bumetanide, spironolactone, amiloride HCl, mannitol, osmofundin, tolvaptan.

^4^Antidiuretics included desmopressin, vasopressin.

^5^Corticosteroids included dexamethasone, fludrocortisone acetate, hydrocortisone, prednisolone.

Significant values are italic.

At ICU admission, a total of 9098 (5.6%), 40,533 (25.0%), and 112,395 (69.4%) patients were hypernatremic, hyponatremic, and normonatremic, respectively. Hypernatremic and hyponatremic patients were older [64.8 (54.0–75.8) years and 65.3 (54.5–75.7) years, respectively] compared with normonatremic patients [63.2 (51.5–73.9) years, P < 0.001], and had higher APACHE IV-predicted risk of death [0.3 (0.1–0.7) and 0.2 (0.1–0.4), respectively; vs 0.1 (0–0.3), P < 0.001]. Patients with hypernatremia and hyponatremia had higher rates of emergency ICU admission [7935 (87.2%) and 36,534 (90.1%), respectively; vs 80,990 (72.1%), P < 0.001], COVID-19 infection [85 (0.9%) and 725 (1.8%), respectively; vs 750 (0.7%), P < 0.001], and sepsis [1,891 (20.8%) and 9239 (22.8%), respectively; vs 13,739 (12.2%), P < 0.001] compared with patients with normonatremia.

### Impact on patient mortality

Unadjusted and adjusted outcomes are shown in Table 2. The overall ICU mortality was 15,533 (9.6%). Patients with hypernatremia and hyponatremia at ICU admission had higher rates of ICU mortality compared with patients with normonatremia [1927 (21.2%) and 4697 (11.6%), respectively; vs 8909 (7.9%), P < 0.001]. Multivariable regression showed that the risks of ICU mortality were higher in patients with hypernatremia and hyponatremia than those with normonatremia upon ICU admission [aOR 1.27, 95% CI 1.19–1.36 and aOR 1.14, 95% CI 1.08–1.19, respectively; P < 0.001 for both vs normonatremia]. A total of 26,508 (16.4%) patients died before hospital discharge. The rates of hospital mortality were also higher in patients with hypernatremia and hyponatremia compared with patients with normonatremia [3,026 (33.3%) and 8207 (20.2%), respectively; vs 15,275 (13.6%), P < 0.001]. The association between serum Na upon ICU admission and increased risks of hospital mortality after adjustment was similar [aOR 1.52, 95% CI 1.43–1.62 and aOR 1.21, 95% CI 1.17–1.26, respectively; P < 0.001 for both vs normonatremia].

**Table 2:** Clinical outcomes of patients with hypernatremia, hyponatremia and normonatremia at the time of ICU admission.

Outcomes	Hypernatremia	P-value	Hyponatremia	P-value	Normonatremia
**ICU mortality**					
Frequency (%)	1927/9098 (21.2%)		4697/40,533 (11.6%)		8909/112,395 (7.9%)
Unadjusted OR (95% CI)	3.12 (2.96–3.30)	< *0.001*	1.52 (1.47–1.58)	< *0.001*	*Reference*
Adjusted OR (95% CI)	1.27 (1.19–1.36)	< *0.001*	1.14 (1.08–1.19)	< *0.001*	
**Hospital mortality**					
Frequency (%)	3026/9098 (33.3%)		8207/40,553 (20.2%)		15,275/112,395 (13.6%)
Unadjusted OR (95% CI)	3.17 (3.02–3.32)	< *0.001*	1.61 (1.57–1.66)	< *0.001*	*Reference*
Adjusted OR (95% CI)	1.52 (1.43–1.62)	< *0.001*	1.21 (1.17–1.26)	< *0.001*	
**ICU length of stay**^1^					
Median (IQR), days	3.4 (1.7–7.6)		2.6 (1.3–5.1)		1.9 (1.0–4.0)
Unadjusted IRR (95% CI)	1.68 (1.65–1.72)	< *0.001*	1.25 (1.24–1.27)	< *0.001*	*Reference*
Adjusted IRR (95% CI)	1.29 (1.26–1.31)	< *0.001*	1.07 (1.06–1.08)	< *0.001*	
**Hospital length of stay**^1^					
Median (IQR), days	23 (10–50)		18 (9–39)		15 (8–33)
Unadjusted IRR (95% CI)	1.41 (1.38–1.44)	< *0.001*	1.03 (1.02–1.04)	< *0.001*	*Reference*
Adjusted IRR (95% CI)	1.25 (1.23–1.28)	< *0.001*	1.03 (1.02–1.04)	< *0.001*	
**Adverse neurological outcome**^2^					
Frequency (%)	253/1154 (21.9%)		920/6428 (14.3%)		22,389/154,444 (14.5%)
Unadjusted OR (95% CI)	1.66 (1.44–1.91)	< *0.001*	0.99 (0.92–1.06)	0.68	*Reference*
Adjusted OR (95% CI)	1.46 (1.26–1.68)	< *0.001*	1.18 (1.09–1.27)	< *0.001*	
**Hospital discharge destination**					
i. Home (use as reference)	3967/9098 (43.6%)		24,394/40,533 (60.2%)		76,909/112,395 (68.4%)
ii. Transfers					
Frequency (%)	2081/9098 (22.9%)		7844/40,533 (19.4%)		19,979/112,395 (17.8%)
Unadjusted OR (95% CI)	2.02 (1.91–2.13)	< *0.001*	1.24 (1.20–1.28)	< *0.001*	*Reference*
Adjusted OR (95% CI)	1.57 (1.48–1.67)	< *0.001*	1.04 (1.01–1.08)	*0.008*	
iii. Death					
Frequency (%)	3028/9098 (33.3%)		8210/40,533 (20.3%)		15,284/112,395 (13.6%)
Unadjusted OR (95% CI)	3.84 (3.65–4.04)	< *0.001*	1.69 (1.64–1.75)	< *0.001*	*Reference*
Adjusted OR (95% CI)	1.85 (1.73–1.97)	< *0.001*	1.23 (1.18–1.28)	< *0.001*	
iv. Others					
Frequency (%)	22/9098 (0.2%)		85/40,533 (0.2%)		226/112,395 (0.2%)
Unadjusted OR (95% CI)	1.89 (1.22–2.93)	*0.005*	1.19 (0.92–1.52)	0.18	*Reference*
Adjusted OR (95% CI)	1.35 (0.87–2.11)	0.18	0.98 (0.76–1.27)	0.90	
**Bed costs**					
Median (IQR), thousand U.S. Dollars	26.7 (13.4–53.6)		21.5 (12.6–40.0)		17.3 (10.6–33.8)
Unadjusted relative cost (95% CI)	1.46 (1.40–1.52)	< *0.001*	1.09 (1.07–1.11)	< *0.001*	*Reference*
Adjusted relative cost (95% CI)	1.25 (1.20–1.29)	< *0.001*	1.04 (1.02–1.06)	*0.001*	

*CI* confidence interval, *ICU* intensive care unit, *IRR* incident rate ratio, *OR* odds ratio.

^1^Reported as incidence rate ratio (IRR).

^2^Analyzed with sever hypernatremia (serum sodium level > 155 mmol/L) and severe hyponatremia (serum sodium level < 125 mmol/L).

Significant values are italic.

### Impact on neurological complications

Adverse neurological outcomes were observed in 23,562 (14.5%) patients and was more frequent in patients with severe hypernatremia (serum Na > 155 mmol/L) and hyponatremia (serum Na < 125 mmol/L) [253 (21.9%) and 920 (14.3%), respectively; vs 22,389 (14.5%), P < 0.001]. Multivariable regression showed that the risks of adverse neurological outcomes were higher in patients with hypernatremia and hyponatremia than those with normonatremia upon ICU admission [aOR 1.46, 95% CI 1.26–1.68 and aOR 1.18, 95% CI 1.09–1.27; P < 0.001 for both vs normonatremia].

### Impact of healthcare service utilization and costs

The median ICU LOS and hospital LOS were 2.0 (1.0–4.6) and 16.0 (8.0–35.0) days, respectively. Patients with hypernatremia and hyponatremia at ICU admission had significantly longer median ICU LOS than those with normonatremia [3.4 (1.7–7.6) days and 2.6 (1.3–5.1) days, respectively; vs 1.9 (1.0–4.0) days, P < 0.001]. In adjusted analysis, patients with hypernatremia or hyponatremia had increased risks for longer ICU LOS [incidence rate ratio (IRR) 1.29, 95% CI, 1.26–1.31 and IRR 1.07, 95% CI 1.06–1.08, respectively; P < 0.001 for both vs normonatremia]. There were similar associations of patients with hypernatremia and hyponatremia with longer median hospital LOS, when compared with patients with normonatremia. Furthermore, patients with hypernatremia and hyponatremia at ICU admission were more likely to be transferred for convalescence care than discharged home compared with patients with normonatremia [hypernatremia: 2,081 (22.9%); relative risk ratio (RRR) 1.57, 95% CI 1.48–1.67; P < 0.001 vs hyponatremia: 7,844 (19.4%); RRR 1.04, 95% CI 1.01–1.08; P = 0.008 vs normonatremia: 19,979 (17.8%)]. The median healthcare cost (in thousand United States (U.S.) dollars) in the cohort was $18.6 (11.3–36.6). Patients with hypernatremia and hyponatremia were associated with higher healthcare costs compared with patients with normonatremia [hypernatremia: $26.7 (13.4–53.6); relative cost 1.25, 95% CI 1.20–1.29; P < 0.001 vs hyponatremia: $21.5 (12.6–40.0); relative cost 1.04, 95% CI 1.02–1.06; P = 0.001 vs normonatremia: $17.3 (10.6–33.8)]. Detailed results on clinical outcomes are presented in Table 2.

### Changes in Na level over time

**Figure 2:**
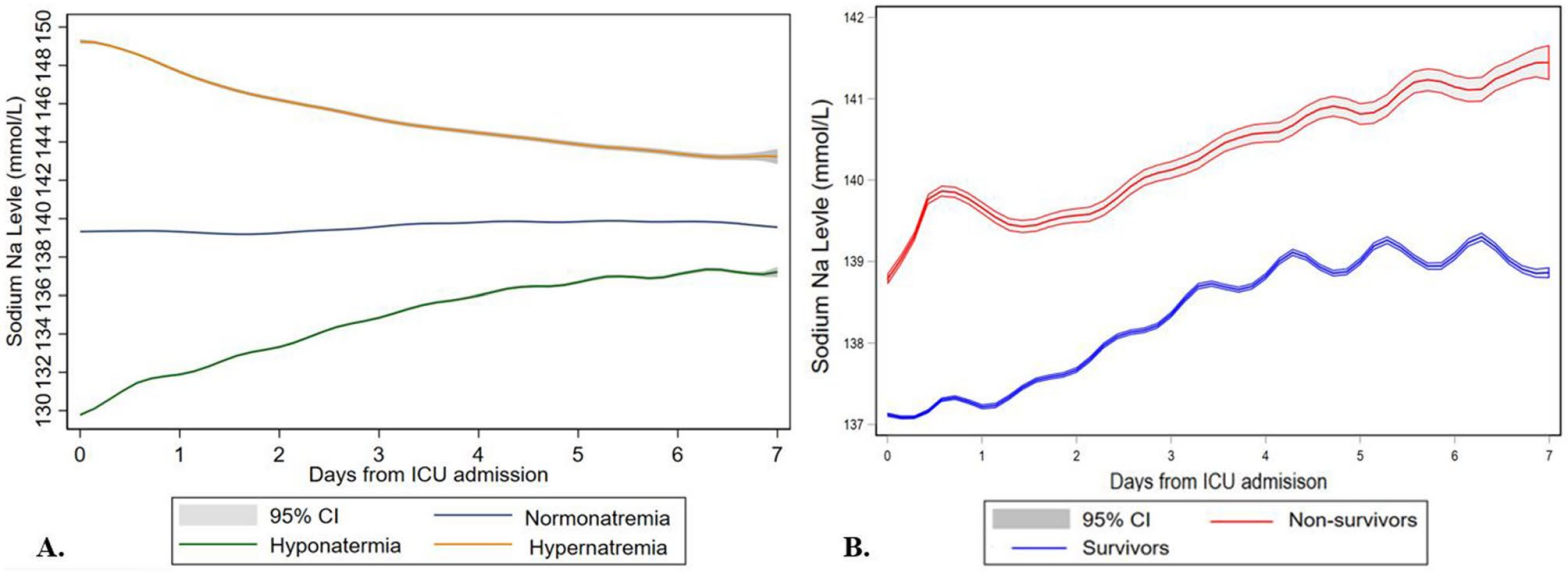
Comparison of serum sodium trends over time. Changes in serum Na level from Day 0 to Day 7 following admission stratified by presence of hypernatremia, hyponatremia, and normonatremia upon ICU admission (**A**), and ICU mortality (**B**). *ICU* intensive care unit, *Na* sodium.

The changes in plasma Na level from Day 0 to Day 7 following ICU admission stratified by exposure groups and ICU mortality are presented in Fig. 2A and B, respectively. The median Na level was lower in ICU survivors compared with ICU non-survivors [138 (135–141) vs. 139 (135–144) mmol/L, P < 0.001].

### Subgroup analyses

The results of subgroup analyses are shown in Table 3. Hypernatremia was associated with higher risks of ICU mortality in both surgical and medical patients (aOR 1.79, 95% CI 1.54–2.07 and aOR 1.13, 95% CI 1.04–1.22, respectively; P < 0.001 for both vs normonatremia). When stratified by the cause of ICU admission, the increased ICU mortality was observed in hypernatremic patients admitted to ICU for both non-neurological and neurological reasons (aOR, 1.24, 95% CI 1.15–1.33 and aOR 1.65, 95% CI 1.29–2.12, respectively; P < 0.001 for both vs normonatremia).

**Table 3:** Subgroup and sensitivity analyses of patients with hypernatremia, hyponatremia and normonatremia at the time of ICU admission.

	Hypernatremia	P-value	Hyponatremia	P-value	Normonatremia
**Subgroup analyses–ICU mortality**					
**Surgical patients**					
Frequency (%)	462/3195 (14.5%)		569/11,321 (5.0%)		1848/55,564 (3.3%)
Unadjusted OR (95% CI)	4.91 (4.41–5.48)	< *0.001*	1.54 (1.40–1.69)	< *0.001*	*Reference*
Adjusted OR (95% CI)	1.79 (1.54–2.07)	< *0.001*	0.87 (0.77–0.98)	*0.026*	
** Medical patients**					
Frequency (%)	1465/5903 (24.8%)		4128/29,212 (14.1%)		7061/56,831 (12.4%)
Unadjusted OR (95% CI)	2.33 (2.18–2.48)	< *0.001*	1.16 (1.11–1.21)	< *0.001*	*Reference*
Adjusted OR (95% CI)	1.13 (1.04–1.22)	< *0.001*	1.17 (1.11–1.23)	< *0.001*	
** Without neurological diseases**					
Frequency (%)	1782/8187 (21.8%)		4557/38,009 (12.0%)		8155/95,594 (8.5%)
Unadjusted OR (95% CI)	2.98 (2.82–3.16)	< *0.001*	1.46 (1.41–1.52)	< *0.001*	*Reference*
Adjusted OR (95% CI)	1.24 (1.15–1.33)	< *0.001*	1.15 (1.10–1.21)	< *0.001*	
**With neurological diseases**					
Frequency (%)	145/911 (15.9%)		140/2524 (5.6%)		754/16,801 (4.5%)
Unadjusted OR (95% CI)	4.03 (3.33–4.88)	< *0.001*	1.25 (1.04–1.50)	*0.018*	*Reference*
Adjusted OR (95% CI)	1.65 (1.29–2.12)	< *0.001*	0.85 (0.68–1.05)	0.14	
**Sensitivity analyses**					
**Redefinition of normal serum sodium**^1^					
Frequency (%)	725/2717 (26.7%)		1726/14,338 (12.0%)		13,082/144,971 (9.0%)
Unadjusted OR (95% CI)	3.67 (3.36–4.00)	< *0.001*	1.38 (1.31–1.46)	< *0.001*	*Reference*
Adjusted OR (95% CI)	1.20 (1.08–1.34)	*0.001*	1.13 (1.06–1.21)	< *0.001*	
**Corrected sodium**^2^					
Frequency (%)	1838/10,728 (17.1%)		2109/16,197 (13.0%)		4808/61,264 (7.9%)
Unadjusted OR (95% CI)	2.43 (2.29–2.57)	< *0.001*	1.76 (1.66–1.86)	< *0.001*	*Reference*
Adjusted OR (95% CI)	1.16 (1.07–1.25)	< *0.001*	1.19 (1.12–1.28)	< *0.001*	

*CI* confidence interval, *ICU* intensive care unit, *OR* odds ratio.

^1^Normonatremia defined as serum sodium level 130–150 mmol/L; hyponatremia defined as serum sodium level < 130 mmol/L; Hypernatremia defined as serum sodium level > 150 mmol/L.

^2^Corrected [Na] = Serum [Na] + 0.4 x (Paired blood glucose–5.5 mmol/L). A total of 73,837 (45.6%) patients did not have corrected sodium measurements.

Significant values are italic.

### Sensitivity analyses

The results of sensitivity analyses are shown in Table 3. After redefining the cut-off value for dysnatremias, serum Na levels upon ICU admission were hypernatremic (serum Na > 150 mmol/L) in 2717 (1.7%), hyponatremic (serum Na < 130 mmol/L) in 14,338 (8.8%), and normal (serum Na 130–150 mmol/L) in 144,971 (89.5%) patients. The effect of dysnatremias on ICU mortality remained robust with the re-defined cut-off values: hypernatremia (aOR 1.20, 95% CI 1.08–1.34; P = 0.001) and hyponatremia (aOR 1.13, 95% CI 1.06–1.21; P < 0.001. Secondly, after adjusting for the effect of hyperglycaemia on serum Na levels, there were 10,728 (12.2%), 16,197 (18.4%), and 61,264 (69.5%) patients with hypernatremia, hyponatremia, and normonatremia, respectively. The association between dysnatremias and ICU mortality remained significant (hypernatremia: aOR 1.16, 95% CI 1.07–1.25; P < 0.001; hyponatremia: aOR 1.19, 95% CI 1.12–1.28; P < 0.001).

Other sensitivity analyses such as examining the use of ICD diagnostic codes to define adverse neurological outcomes are included in the Supplementary Sect. 1.3 online.

### Exploratory analysis

The results of exploratory analyses are shown in Table S1 online. The effect of dysnatremias on ICU mortality was significantly modified by DM in patients with hypernatremia (P for interaction = 0.003). Hypernatremia was associated with increased ICU mortality in patients without DM (aOR 1.34, 95% CI 1.23–1.44; P < 0.001) but insignificantly associated with ICU mortality in patients with DM (aOR 1.05, 95% CI 0.90–1.23; P = 0.55).

## Discussion

Dysnatremias are common and important electrolyte abnormalities in critically ill patients. Our data suggested that dysnatremias are prevalent among critically ill patients at the time of ICU admission. Importantly, the presence of hypernatremia or hyponatremia early in the course of critical illness were both associated with various adverse clinical outcomes including increased ICU and in-hospital mortality, neurological complications, longer ICU and hospital stay, worse discharge outcomes, and consequentially higher healthcare costs.

The incidences of hypernatremia and hyponatremia reported in previous series were variable, ranging from 0.5–9.0% and 13.7–34.4%, respectively^1–3,8,12–16^. In this large multicenter cohort of mixed surgical and medical ICU patients, dysnatremias occurred in 30.6% of patients upon ICU admission, with rates of hypernatremia and hyponatremia at 5.6% and 25.0%, respectively. The discrepancy in the frequency of dysnatremias reported in different studies may be explained by the differences in case mix and cut-off values for normal Na level. The incidences for dysnatremias were higher in studies that included primarily medical or mixed medical-surgical cohorts, suggesting that the burden of pre-existing medical comorbidities may predispose to dysnatremias in critically ill patients. Indeed, patients with dysnatremias in our study were older and had higher APACHE IV scores and rates of sepsis compared to those with normonatremia. Notably, there is a temporal shift to growing proportion of hypernatremia over time^7^, possibly a result of changes in critical care practices such as fluid management^17,18^. The rise in proportion of patients with hypernatremia in the ICU has paralleled a growth of ICU admissions in many developed countries^6^.

Mounting evidence have demonstrated the impact of dysnatremias on ICU mortality, even after adjustment for case mix and severity of illness^1,2,8,19^. Moreover, the risk of death escalated with the magnitude of the deviation from normal values^20^. One should also note that even if these patients survived their ICU stay, the overall in-hospital mortality remained alarmingly high (33.3% and 20.2% in patients with hypernatremia and hyponatremia, respectively). Furthermore, hypernatremia appeared to have a greater impact on clinical outcomes compared with hyponatremia. In our cohort, patients with hypernatremia at ICU admission had a 27% increased odds of ICU mortality and 52% increased odds of hospital mortality compared to patients with normonatremia. Indeed, hypernatremia, often an indication of volume depletion, is associated with hypotension, hypoperfusion, and subsequent increased mortality in patients with fulminant sepsis or after major operations^18^. Our finding that hypernatremia conveys an increased risk of mortality in surgical and neurologic patients echoes previous findings in liver transplantation and neurosurgical cohorts^21,22^. On the contrary, hyponatremia often occurs in ICU patients who are fluid overloaded and occasionally in those with syndrome of inappropriate antidiuretic hormone due to severe chest infection, such as recently reported in COVID-19 disease^5^.

The pathophysiology of dysnatremias in increasing patient mortality remains to be thoroughly examined. Putative mechanisms for dysnatremias (especially hypernatremia) to increase mortality include its effects on the neurological, cardiovascular and immunological systems. On a cellular level, Na plays an important role in maintaining tonicity in body compartments and providing optimal enzymatic conditions for essential metabolic processes. Hypernatremia causes serum hyperosmolality, which impairs normal metabolism and transmission of nerve signals. Hypernatremia also decreases cardiomyocyte contractility, thereby compromising cardiac output^23,24^. There is also evidence from animal studies that osmotic stress impairs immune function by affecting activation and differentiation of B lymphocytes, which are key effector cells for fighting against infections^25^. Further complicating the situation are the competing priorities for treatment in patients who were admitted to ICU for other conditions but had hypernatremia at presentation, as fluid therapy may contradict with the management strategy of concurrent illnesses. The strong association between dysnatremias at the time of ICU admission and mortality highlights the importance of proper fluid and electrolyte management when patients are managed at the emergency department, general medical/surgical wards, or during the peri-/intra-operative periods. In this study, we observed that hypernatremia was associated with increased ICU mortality in patients without diabetes but not in diabetic subjects. The finding was intriguing but it is possible that diabetic patients have many other medical co-morbidities and impaired immune responses that attenuated the effect of dysnatremias on mortality.

Neurological sequelae are important complications of dysnatremias, and are attributable to the Na disturbance per se or overzealous correction. It is well recognized that the tempo of Na correction is important as overly rapid normalization can lead to seizures and potentially life-threatening complications such as cerebral oedema or central pontine demyelination^26–28^. In this study, despite steady correction of the Na level over 75 h in most patients with dysnatremias, up to 15% of patients had evidence of adverse neurological events. Hypernatremia appeared to confer higher risk of subsequent neurological complications compared to hyponatremia. Disturbance in osmoregulation, abnormal thirst sensation and aberrant anti-diuretic hormone response all exacerbate the effects of hypernatremia. The use of mannitol in patients with raised intracranial pressure may also precipitate a hypertonic milieu for neuronal cells^29^. While previous studies mostly reported the effect of hypernatremia in ICU patients admitted for neurological causes, we showed that hypernatremia at the time of ICU admission was predictive of adverse neurological sequelae in patients with non-neurological conditions. Such finding calls for closer neurological monitoring in ICU patients admitted for non-neurological causes who had abnormal Na levels at the time of admission.

Apart from affecting mortality and neurological outcomes, the impact of dysnatremias also translated to longer duration of stay in ICU and hospital, as well as higher healthcare costs. The burden on healthcare resources are related to the metabolic and neurological sequelae of dysnatremias, which often require prolonged duration on life support and more convoluted process of recovery. Indeed, the downstream effects of adverse neurological outcomes were exemplified by a 50% increase in the requirement for rehabilitation before discharge in patients with hypernatremia at the time of ICU admission.

One limitation was the retrospective nature of this study, which may lead to potential biases in other aspects of patient management and problems of residual confounding. For instance, data on fluid management and relevant intervention after ICU admission were not systematically recorded. The measurement of secondary outcomes such as adverse neurological outcomes were not prospectively recorded and may be subjected to errors in coding. It was also not possible to quantitatively gauge the severity of neurological impairment. Lastly, healthcare costs were estimated by the average cost of different types of hospital beds, and outliers in cost of management such as requirement for advanced life support and expensive medications may lead to over/under-estimation of cost data. Notwithstanding, our study included territory-wide data of almost all patients admitted to ICUs across a ten-year study period. The large study population (162,026 patients) with mixed medical/surgical cohorts and few exclusion criteria allowed us to conduct relevant stratified analyses. Our findings are therefore representative of real-world data and are highly generalizable to other ICU cohorts.

Dysnatremias are common in critically ill patients at the time of ICU admission, and are robust predictors for mortality, neurological complications and increased healthcare costs.

### Supplementary Information

Supplementary Information.

## Data Availability

The datasets used and/or analysed during the current study are available from the corresponding author on reasonable request.

## Abbreviations

APACHE IV: Acute physiology and chronic health evaluation IV
CDARS: Clinical data analysis and reporting system
CI: Confidence interval
DM: Diabetes mellitus
ICD-9-CM: International classification of diseases, 9th revision, clinical modification
ICU: Intensive care unit
IQR: Interquartile range
IRR: Incidence rate ratio
LOS: Length of stay
OR: Odds ratio
