# Supplementary material for: A retrospective cohort study on the clinical outcomes of patients admitted to intensive care units with dysnatremia
Source: Sci Rep. 2023 Dec 1;13:21236. doi: 10.1038/s41598-023-48399-5 (PMC10692105; doi:10.1038/s41598-023-48399-5)

**A Retrospective Cohort Study on the Clinical Outcomes of Patients Admitted to Intensive Care Units with Dysnatremia**

**Supplementary Materials**

**Table of content**

| **Materials** | **Pages** |
| --- | --- |
| **Section 1.1** List of comorbidities defined by International Classification of Diseases, 9^th^ Revision, Clinical Modification (ICD-9-CM) | 2 |
| **Section 1.2**  List of APACHE disease categories used to define admission for neurologic disease | 3-4 |
| **Section 1.3** Adverse neurological outcomes defined by International Classification of Diseases, 9^th^ Revision, Clinical Modification (ICD-9-CM) | 5 |
| **Method.** Kernel-weighted local polynomial regression for average sodium levels during first 7 days of ICU admission | 6 |
| **Table S1.** Exploratory analyses – Effect of diabetes on the association between dysnatremia and ICU mortality | 7-8 |
| **Table S2.** Exploratory analyses – Effect of age and APACHE risk of death on the association between dysnatremia and ICU mortality | 9 |
| **Table S3.** Temporal trends in normonatremia, hypernatremia and hyponatremia from 2010 to 2022 | 10 |
| **Figure S1.** Restricted cubic spline plot of serum sodium levels upon ICU admission and risks of ICU mortality | 11 |

**Section 1.1** List of comorbidities defined by International Classification of Diseases, 9^th^ Revision, Clinical Modification (ICD-9-CM)

| **Comorbidities** | **ICD-9-CM Diagnostic codes** |
| --- | --- |
| Cardiovascular diseases | 390-429 |
| Diabetes mellitus | 250 |
| Cerebrovascular diseases | 430-438 |
| Respiratory diseases | 490-496 |
| Malignancies | 140-239 |
| Kidney diseases | determined by eGFR <15 ml/min/1.73 m^2^ using 2021 CKD-EPI equation ^1^ |
| Liver diseases | 571-572 |
| Dementia | 290 |
| COVID-19 | 519.8(8), 647.60(17), 647.61(2), 647.63(4), 647.64(1) |

**References:**

1 Inker, L. A. *et al.* New Creatinine- and Cystatin C-Based Equations to Estimate GFR without Race. *N Engl J Med.* **385**, 1737-1749 (2021).

**Section 1.2** List of APACHE disease categories used to define admission for neurologic disease

| **Disease Category Sub code** | **Description** |
| --- | --- |
| 1503.01 | Arteriovenous malformation, surgery for |
| 1503.02 | Subarachnoid haemorrhage/Intracranial aneurysm, surgery for |
| 1504.01 | Complications of previous spinal cord surgery, surgery for |
| 1504.02 | Devices for spine fracture/dislocation |
| 1504.03 | Fusion-spinal/Harrington rods |
| 1504.04 | Neoplasm-spinal cord surgery or other related procedures |
| 1504.05 | Spinal cord surgery, other |
| 1505.01 | Neoplasm-cranial, surgery for (excluding transphenoidal) |
| 1505.02 | Transphenoidal surgery |
| 1506.01 | Abscess/Infection-cranial, surgery for |
| 1506.02 | Anastomosis, vascular |
| 1506.03 | Biopsy, brain |
| 1506.04 | Burr hole placement |
| 1506.05 | Cerebrospinal fluid leak, surgery for |
| 1506.06 | Cranioplasty and complications from previous craniotomies |
| 1506.07 | Neurologic surgery, other |
| 1506.08 | Seizures-intractable, surgery for |
| 1506.09 | Shunts and revisions |
| 1506.1 | Stereotactic procedure |
| 1506.11 | Ventriculostomy |
| 401.01 | Haemorrhage/haematoma, intracranial |
| 402.01 | Subarachnoid haemorrhage/arteriovenous malformation |
| 402.02 | Subarachnoid haemorrhage/intracranial aneurysm |
| 403.01 | CVA, Cerebrovascular accident/stroke |
| 404.01 | Abscess, neurologic |
| 404.02 | Encephalitis |
| 404.03 | Meningitis |
| 405.01 | Neoplasm, neurologic |
| 406.01 | Amyotrophic lateral sclerosis |
| 406.02 | Guillian-Barre syndrome |
| 406.03 | Myasthenia gravis |
| 406.04 | Neuromuscular medical, other |
| 407.01 | Seizures (primary-no structural brain disease) |
| 408.01 | Hydroceph0alus, obstructive |
| 408.02 | Neurologic medical, other |
| 408.03 | Palsy, cranial nerve |
| 409.01 | Haematoma, epidural |
| 409.02 | Haematoma, subdural |
| 410.01 | Coma/change in level of consciousness (not hepatic, diabetic or CA related) |

**Section 1.3** Adverse neurological outcomes defined by International Classification of Diseases, 9^th^ Revision, Clinical Modification (ICD-9-CM)

| **Diseases** | **ICD-9-CM Diagnostic codes** |
| --- | --- |
| Inflammatory diseases of the central nervous system | 320 to 326 |
| Hereditary and degenerative disease of the central nervous system | 330 to 337 |
| Other disorders of the central nervous system | 340 to 349 |
| Disorders of the peripheral nervous system | 350 to 359 |

As a sensitivity analysis, we examined the effects of defining adverse neurological outcomes using ICD9 diagnostic codes. A total of 15,079 (9.3%) patients had adverse neurological outcomes. The odds of adverse neurological outcomes for patients with hypernatremia remained significantly higher compared to those with normonatremia (adjusted odds ratio 1.29, 95%CI 1.09 – 1.53, P=0.003).

**Method.** Kernel-weighted local polynomial regression for average sodium levels during first 7 days of ICU admission

In Figure 2, the Y-axis is serum sodium level and the X-axis is time from ICU admission. We used Kernel-weighted local polynomial regression to create smooth curves for the ‘normal’, ‘hypernatremia’ and ‘hyponatremia’ groups. Kernel-weighted local polynomial regression estimates the value of the curve at each point based on nearby data points. The weight of each nearby point is determined using a kernel function, which assigns higher weights to points that are closer and lower weights to points that are farther away. Once the weights are assigned, a polynomial function is fitted to the nearby points.

| **Table S1.** Exploratory analyses – Effect of diabetes on the association between dysnatremia and ICU mortality | | | | | |
| --- | --- | --- | --- | --- | --- |
|  | **Hypernatremia** | **P-value** | **Hyponatremia** | **P-value** | **Normonatremia** |
| **P-value for ‘dysnatremia x diabetes’** | *0.003* |  | 0.26 |  | *Reference* |
|  |  |  |  |  |  |
| ***With Diabetes*** | | | | |  |
| Frequency (%) | 354 / 1,733 (20.4%) |  | 1,258 / 12,053 (10.4%) |  | 1,772 / 20,492 (8.7%) |
| Unadjusted OR (95% CI) | 2.71 (2.39 - 3.08) | *<0.001* | 1.23 (1.14 - 1.33) | *<0.001* | *Reference* |
| Adjusted OR (95% CI) | 1.05 (0.90 - 1.23) | 0.55 | 1.10 (1.00 - 1.21) | *0.043* |  |
|  |  |  |  |  |  |
| ***Without Diabetes*** | | | | |  |
| Frequency (%) | 1,573 / 7,365 (21.4%) |  | 3,439 / 28,480 (12.1%) |  | 7,137/ 91,903 (7.8%) |
| Unadjusted OR (95% CI) | 3.23 (3.04 - 3.43) | *<0.001* | 1.63 (1.56 - 1.70) | *<0.001* | *Reference* |
| Adjusted OR (95% CI) | 1.34 (1.23 - 1.44) | *<0.001* | 1.14 (1.08 - 1.21) | *<0.001* |  |
| **Abbreviation:** CI – confidence interval; OR - odds ratio.  The effect of diabetes on the association between dysnatremia and ICU mortality was examined by introducing an interaction term ‘dysnatremia x diabetes’ to the regression model. The results showed that hypernatremia was significantly associated with increased ICU mortality in patients without diabetes (adjusted odds ratio [aOR] 1.34, 95% CI 1.23-1.44, P<0.001) but insignificantly associated with ICU mortality in patients with diabetes (aOR 1.05, 95% CI 0.90-1.23; P=0.55) (P for interaction = 0.003). | | | | | |
|  |  |  |  |  |  |

**Table S2.** Subgroup analyses – Effect of age and APACHE risk of death on the association between dysnatremia and ICU mortality

| **Outcome:**  **ICU mortality** | **Hypernatremia** | **P-value** | **Hyponatremia** | **P-value** | **Normonatremia** |
| --- | --- | --- | --- | --- | --- |
| **P-value for ‘dysnatremia x Age’** | *<0.001* |  | *<0.001* |  | *Reference* |
|  |  |  |  |  |  |
| **Age <65 years** |  | | | | |
| Frequency (%) | 813 / 4,587  (17.7%) |  | 1,999 / 19,960  (10.0%) |  | 3,577 / 61,369  (5.8%) |
| Adjusted odds ratio  (95% CI) | 1.48  (1.32 – 1.65) | *<0.001* | 1.27  (1.18 – 1.37) | *<0.001* | *Reference* |
|  |  |  |  |  |  |
| **Age ≥65 years** |  | | | | |
| Frequency (%) | 1,114 / 4,511  (24.7%) |  | 2,698 / 20,573  (13.1%) |  | 5,332 / 51,026  (10.5%) |
|  |  |  |  |  |  |
| Adjusted odds ratio  (95% CI) | 1.15  (1.05 – 1.25) | *0.003* | 1.05  (0.99 – 1.11) | 0.14 | *Reference* |
|  |  |  |  |  |  |
|  |  |  |  |  |  |
| **P-value for ‘dysnatremia x APACHE risk of death’** | *0.011* |  | 0.79 |  | *Reference* |
|  |  |  |  |  |  |
| **APACHE risk of death <0.12** | | | | | |
| Frequency (%) | 25 / 2,564  (1.0%) |  | 82 / 15,428  (0.5%) |  | 264 / 62,879  (0.4%) |
|  |  |  |  |  |  |
| Adjusted odds ratio  (95% CI) | 1.78  (1.18 – 2.71) | *0.007* | 0.88  (0.68 – 1.14) | 0.33 | *Reference* |
|  |  |  |  |  |  |
|  |  |  |  |  |  |
| **APACHE risk of death ≥0.12** | | | | | |
| Frequency (%) | 1,902 / 6,534  (29.1%) |  | 4,615 / 25,105  (18.4%) |  | 8,645 / 49,516  (17.5%) |
|  |  |  |  |  |  |
| Adjusted odds ratio  (95% CI) | 1.22  (1.14 – 1.31) | *<0.001* | 1.10  (1.05 – 1.16) | *<0.001* | *Reference* |

**Abbreviation:** CI – confidence interval.

**Table S3.** Temporal trends in normonatremia, hypernatremia and hyponatremia from 2010 to 2022 ICU year

| **ICU year** | **Hypernatremia**  **(Na > 155 mmol/L)** | **Hyponatremia**  **(Na < 125 mmol/L)** | **Normonatremia** |
| --- | --- | --- | --- |
| 2010 | 77 (0.7%) | 414 (3.8%) | 10,533 (95.6%) |
| 2011 | 57 (0.5%) | 419 (3.7%) | 11,017 (95.9%) |
| 2012 | 88 (0.7%) | 450 (3.7%) | 11,724 (95.6%) |
| 2013 | 89 (0.8%) | 356 (3.0%) | 11,356 (96.2%) |
| 2014 | 86 (0.8%) | 348 (3.3%) | 10,252 (95.9%) |
| 2015 | 78 (0.6%) | 454 (3.6%) | 12,255 (95.8%) |
| 2016 | 87 (0.7%) | 524 (4.0%) | 12,515 (95.4%) |
| 2017 | 79 (0.6%) | 568 (4.2%) | 13,021 (95.3%) |
| 2018 | 91 (0.7%) | 514 (3.7%) | 13,277 (95.6%) |
| 2019 | 121 (0.8%) | 559 (3.9%) | 13,762 (95.3%) |
| 2020 | 107 (0.8%) | 640 (4.5%) | 13,528 (94,8%) |
| 2021 | 106 (0.7%) | 652 (4.3%) | 14,596 (95.1%) |
| 2022 | 88 (1.2%) | 530 (7.3%) | 6,658 (91.5%) |

**Figure S1.** Restricted cubic spline plot of serum sodium levels upon ICU admission and risks of ICU mortality

The X-axis is the serum sodium level upon ICU admission, while the Y-axis is the adjusted odds ratio for the primary outcome of ICU mortality. Between admission sodium levels of 120 to 160 mmol/L, we examined possible nonlinear associations by modeling admission serum sodium using a restricted cubic spline with 5 knots located at the 5th, 25th, 50th, 75th, and 95th percentiles of the distribution.


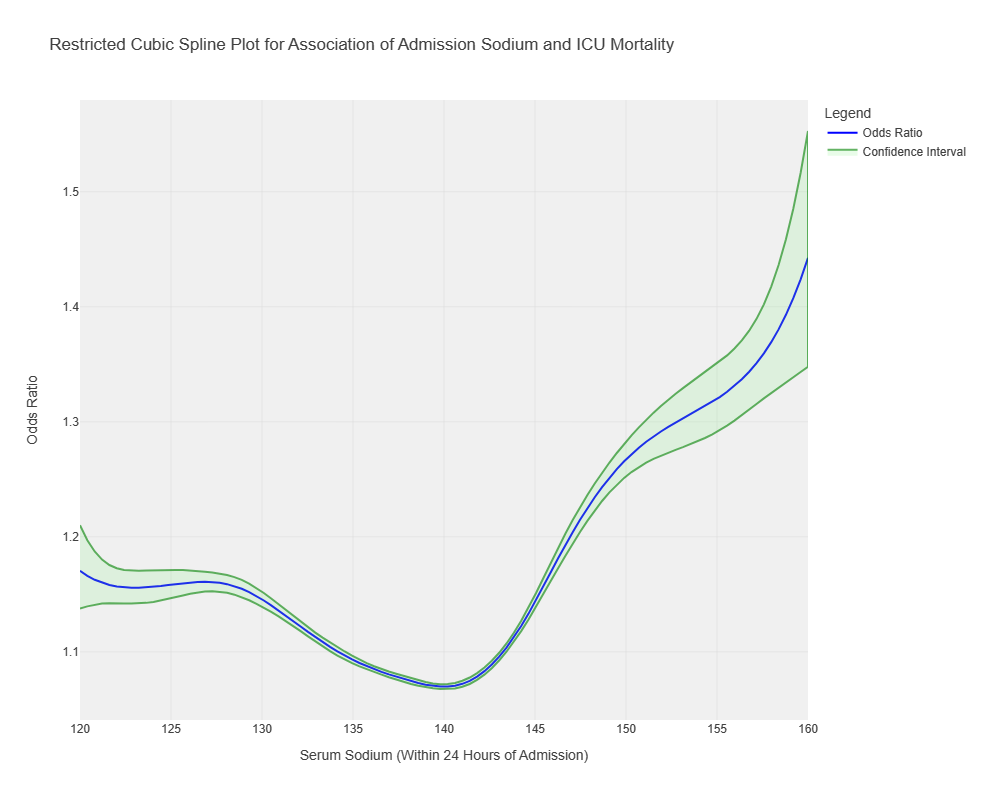

Supplement: Supplementary file 1 — Supplementary Information. [file 41598_2023_48399_MOESM1_ESM.docx]
